# Two studies of Tsarang village, Upper Mustang Nepal with different results

**DOI:** 10.1186/s40101-025-00405-7

**Published:** 2025-10-06

**Authors:** Sienna R Craig, Anna Di Rienzo, Frank L Powell, Kingman P Strohl, Cynthia M Beall

**Affiliations:** 1 Anthropology, Dartmouth College, Hanover, NH 03755 US; 2 Human Genetics, University of Chicago, Chicago, IL 60637 US; 3 Division of Pulmonary, Critical Care, Sleep Medicine and Physiology, Department of Medicine, University of California San Diego, La Jolla, CA 92023 US; 4 Division of Pulmonary, Critical Care and Sleep Medicine, School of Medicine, Case Western Reserve University, Cleveland, OH 44106 US; 5 Anthropology, Case Western Reserve University, Cleveland, OH 44106 US

**Keywords:** Adaptation, Biological, Hypoxia, Tibetans, Data reporting, Obesity, Hypertension, Polycythemia, *EPAS1*

## Abstract

The recent *Journal of Physiological Anthropology *article on polycythemia among Tibetan highlanders (Arima et al., J Physiol Anthropol 43:25, 2024) piqued our interest because we collected similar data in the same Nepali village in Upper Mustang two years later with notably different results (Cho et al., Evol Med Public Health 2017:82–96, 2017; Ye et al., Proc Natl Acad Sci U S A 121:e2403309121, 2024). Arima et al report high prevalences of chronic disease and conclude that Tsarang villagers have poor health. Here, we describe our relevant findings to show that authors' definitions and other research design elements can yield different population health implications. Our study sampled ethnic Tibetan Upper Mustang women 39 Years and older in 2012 who had been married or pregnant and were lifelong residents of this village at 3500m. At our 2019 follow-up study, the women were 46 Years and older. Fifty-five of the 64 eligible Tsarang residents (85%) participated in 2019 study designed to examine the influences of genes and physiology on reproductive success. Arima et al. sampled all Tsarang residents 18 or older in 2017, therefore, our studies include many of the same women in their mid-40s and older. Arima et al reported that 12% of the sample were polycythemic, whereas we found none; they reported 26% obesity whereas we found none; they showed 17% of the sample had hypertension, whereas we found 27%. Factors that may account for the differences in estimates of chronic disease prevalence in Tsarang include age differences in the samples, a wider age range in the currently reported sample, undefined cut-off values for disease categories, while we applied and reported chronic diseases using standard definitions. Because our study did not replicate the findings of Arima et al., we caution against concluding that women in Tsarang have alarming rates of obesity, polycythemia, and hypoxia. Our studies agree that high blood pressure is a public health problem among women in Tsarang. Future use of clear definitions of disease categories will help establish a common understanding of a population’s health.

The recent *Journal of Physiological Anthropology* article on polycythemia among Tibetan highlanders [1] piqued our interest because we collected similar data in the same Nepali village in Upper Mustang 2 years later with very different results [2, 3]. Here, we describe our relevant findings to show that the authors’ definitions and other research design elements can yield different population health implications.

Our study sampled ethnic Tibetan Upper Mustang women 39 Years and older in 2012 who had been married or pregnant and were lifelong residents of this village at 3500m. At our 2019 follow-up study, the women were 46 Years and older. Fifty-five of the 64 eligible Tsarang residents (85%) participated in 2019 study designed to examine the influences of genes and physiology on reproductive success. Arima et al. sampled all Tsarang residents 18 or older in 2017, therefore, our studies include many of the same women in their mid-40s and older.

To see if our data replicate the findings of Arima et al., we apply their definitions of ill health to the data provided by the 55 Tsarang women in our sample. We found 13% overweight (BMI ≥ 25 and < 30 kg/m^2^) and no obese (BMI ≥ 30 kg/m^2^) women (Table 1), compared with 26% obesity and overweight reported by Arima et al. 27% of the women in our sample had hypertension compared with 17% reported by Arima et al. Systolic, but not diastolic, blood pressure correlated with older age in our sample (r_age-Systolic_ = + 0.309 *p* < 0.001; r_age_-r_Diastolic_ = + 0.006, *p* = 0.903) and may partly explain the higher prevalence of hypertension in our 2019 sample. Arima et al. do not reference the cut-offs used; they may have adopted widely-used World Health Organization criteria for obesity, overweight, and hypertension [4, 5].

**Table 1:** Prevalence of Chronic Conditions among ethnic Tibetan women aged 46 –86 Years residing in Tsarang, Mustang District, Nepal, who participated in a 2019 study by the authors (*n*=55, 86% of eligible women from Tsarang) using the categories defined by Arima et al. [1] to define chronic disease

Trait		Age yrs	BMI kg/m^2^	% O_2_ saturation	Systolic Blood Pressure mm Hg	Diastolic Blood Pressure mm Hg	Hemoglobin concentration, gm/dL
**Continuous Variables**							
Mean		59.8	21.4	87.7	122.8	81.5	13.4
Standard Deviation		9.92	3.33	4.0	23.3	13.5	1.3
Minimum		46	13.68	74.00	86.00	57.33	8.40
Maximum		84	29.16	94.00	218.00	132.67	15.73
**Categorical Variables Describing Chronic Disease using Arima et al. definitions, % (N)**							
% Overweight or Obese			12.7% (7)				
% Hypoxemic				67.3% (37)			
% Hypertensive					27.3% (15)		
% Systolic Hypertension					23.6% (13)		
% Diastolic Hypertension						20.0% (11)	
% Polycythemic							0

The World Health Organization does not define hypoxia or polycythemia. Arima et al. should provide their references or logic when choosing cut-off values. These two outcomes commonly used in studies of people under the stress of high-altitude hypoxia—the percent of oxygen saturation of hemoglobin and hemoglobin concentration—differ substantially between their study and ours. Arima et al. measured hypoxia using the percent of arterial oxygen saturation of hemoglobin and reported that 28% of the women in their sample were hypoxic. Everyone in Tsarang experiences environmental hypoxia owing to residence at 3560 m; some may experience additional hypoxemia owing to illness or older adult age (r_age-O2sat_ = −0.367 *p* < 0.001 in our comparator sample of 55 Tsarang women). Without providing justification, Arima et al. Chose a 90% cut-off for hypoxia. That cut-off is above the mean for our sample (Table 1) and categorizes 67% of our sample as hypoxic and, in their view, chronically ill. The inability to distinguish environmental from pathological sources of hypoxia and the lack of a functional definition renders their classification of hypoxia uninterpretable as a measure of chronic disease. It makes no sense to infer disease states from environmental hypoxia.

As for hemoglobin concentration, studies of high-altitude residents use the concept of excess erythrocytosis—a hemoglobin concentration above 19 gm/dL for women [6]. Arima et al. used a hemoglobin concentration above 16 gm/dL as the definition for the related concept of polycythemia (too many red blood cells (McMullin2014)). In contrast to the 12% reported by Arima et al., none of the women in our sample had hemoglobin concentration above 16 gm/dL, that is, no excess erythrocytosis or polycythemia according to conventional or Arima et al. definitions.

Regarding candidate gene analysis, Arima et al. genotyped one single nucleotide polymorphism (SNP), rs2790859, described as an *EGLN1* SNP. It lies outside of the selection signal in *EGLN1*; in fact, it is roughly 43 kb upstream of the gene. The authors did not explain why they chose that candidate SNP. Our analyses of SNPs in the selection signal did not replicate their female-specific association for *EGLN1* with lower Hb. Arima et al. also genotyped a candidate SNP in the selection signal in EPAS1, which our analyses also detected. Our candidate SNP analysis of a different *EPAS1* SNP (rs76242811) confirmed an association with hemoglobin concentration [3].

What might account for the 10–39% differences in estimates of chronic disease prevalence in Tsarang? Differences between the two studies may be partly due to the presence or absence of 18–45 Year olds. Arima et al. do not report the full age range of their sample of people 18 and older; our sample did not include people between 18 and 45. Our finding that BMI declined with age (r _age-BMI_ = −0.153, *p* < 0.002) may partly explain less overweight in our sample. Yet the different age ranges do not account for all the failures to replicate. Our finding that hemoglobin concentration did not correlate with age (r_age_-_Hb_ = −0.065, *p* = 0.191) shows that the age differences do not explain the absence of polycythemia in our samples. Methodological contrasts may contribute to the different results. We measured height without shoes and corrected weight for clothing [3]; Arima et al. do not explain how they collected those data. Finally, differences between the two studies may arise from Arima et al.’s use of idiosyncratic cut-offs that lack support from evidence-based standards.

## Conclusions

In summary, because our study did not replicate the findings of Arima et al., we caution against concluding that women in Tsarang have alarming rates of obesity, polycythemia, and hypoxia. Our studies agree that high blood pressure is a public health problem among women in Tsarang. These findings demonstrate how different definitions and research design elements influence conclusions. Future use of clear definitions of disease categories will help establish a common understanding of a population’s health.

## Data Availability

The datasets generated and/or analysed during the current study are available in the Dryad repository (genetic data) (https://datadryad.org/stash/dataset/doi:10.5061/dryad.bp46m) (https:/datadryad.org/stash/dataset/doi:10.5061/dryad.bp46m) and the Open Science repository (https://osf.io/24fyn/?view_only=edb1fa7c2ded4bc2bd1f4a23003e4618) (https:/osf.io/24fyn/?view_only=edb1fa7c2ded4bc2bd1f4a23003e4618) (phenotypic data).

## Abbreviations

BMI: Body mass index (weight in kg divided by height in meters squared)
Kg: Kilogram
M: Meter
Sat: Percent of oxygen saturation of hemoglobin
Hb: Hemoglobin concentration, gm/dL
SNP: Single nucleotide polymorphism
*EGLN1*: Gene name for Egl-9 Family Hypoxia Inducible Factor 1
*EPAS1*: Gene name for Endothelial PAS Domain Protein 1
